# Complex Approach to Conceptual Design of Machine Mechanically Extracting Oil from *Jatropha curcas *L. Seeds for Biomass-Based Fuel Production

**DOI:** 10.1155/2016/7631458

**Published:** 2016-09-07

**Authors:** Ivan Mašín, Michal Petrů

**Affiliations:** Institute for Nanomaterials, Advanced Technologies and Innovation, Technical University of Liberec, 46117 Liberec, Czech Republic

## Abstract

One of important sources of biomass-based fuel is* Jatropha curcas *L. Great attention is paid to the biofuel produced from the oil extracted from the* Jatropha curcas *L. seeds. A mechanised extraction is the most efficient and feasible method for oil extraction for small-scale farmers but there is a need to extract oil in more efficient manner which would increase the labour productivity, decrease production costs, and increase benefits of small-scale farmers. On the other hand innovators should be aware that further machines development is possible only when applying the systematic approach and design methodology in all stages of engineering design. Systematic approach in this case means that designers and development engineers rigorously apply scientific knowledge, integrate different constraints and user priorities, carefully plan product and activities, and systematically solve technical problems. This paper therefore deals with the complex approach to design specification determining that can bring new innovative concepts to design of mechanical machines for oil extraction. The presented case study as the main part of the paper is focused on new concept of screw of machine mechanically extracting oil from* Jatropha curcas *L. seeds.

## 1. Introduction

The use of bioenergy as energy derived from biofuels in the world permanently increases [1, 2]. Biomass-based fuels as renewable organic source of bioenergy have advantages (e.g., no harmful carbon dioxide emissions, reduction of dependency on fossil fuels, and versatility) and some disadvantages (e.g., requiring more land, relative ineffectiveness when compared to gasoline, and problematic supply chain) as well [3–6]. One of important sources of biomass-based fuel is* Jatropha curcas *L. [7–10].* Jatropha curcas *L. is crop with inconsiderable potential due to its high oil content, rapid growth, easy propagation, drought tolerant nature, ability to grow and reclaim various types of land, need for less irrigation and less agricultural inputs, pest resistance, short gestation periods, and suitable traits for easy harvesting enumerated [11]. Biooil extracted from* Jatropha curcas *L. seeds has positive chemical properties (e.g., better oxidative stability compared to soybean oil, lower viscosity than castor oil, and lower pour point than palm oil) [12].* Jatropha* significant advantage is that it is one of the cheapest sources for biodiesel production (compared to palm oil, soybean, or rapeseed) [13]. On the other hand former and recent findings [14–17] also show that researchers, economists, biochemists, farmers, machine designers, and biofuel producers should not just automatically follow the initial* Jatropha* hype but critically reflect on, for example, current economic situation, state biofuel policy, institutional factors, labour costs, water irrigation, local differences, and last but not least farmer's needs. The evaluations [15], for example, opened many questions connecting with* Jatropha* processing profitability. One of the recommendations in [15] mentioned mechanised extraction as the most efficient and feasible method for oil extraction for small-scale farmers. Consequently one of the strategies of how to produce biofuel from* Jatropha curcas *L. in more efficient manner is to increase the effectiveness of oil pressing process, which would increase the benefits of small-scale farmers. This objective can be achieved through the further innovations of mechanical expellers or presses for small-scale farmers. Due to the great attention paid to this issue [18–26] innovators should be aware that further* Jatropha*-presses development is possible only when applying the systematic approach and design methodology in all stages of engineering design as an essential part of* Jatropha*-press life-cycle. Systematic approach in this case means that designers and development engineers rigorously apply scientific knowledge, integrate different constraints and user priorities, carefully plan product and activities, and systematically solve technical problems. Basic phases of the engineering design process have been in the past developed into more detailed procedures focused on the systematic development [27–33], on creative solution of technical problems [34–37], or on the preliminary and detailed embodiment design [38–40]. The correct definition of the right problem in the form of design specifications is widely regarded as a decisive step towards the effective implementation of all engineering design procedures [41–43]. Two information transformations are required to determine design specification. During the first information transformation the user's needs are translated to functional requirements. The second information transformation takes place when converting the functional requirements to machine characteristics (design specifications) that have been selected to ensure fulfilment of specified functional requirements. By performing these transformations design assignment is then defined as an information input to concept generation phase and subsequent detailed designing. During this process various methods such as marketing research [44, 45], voice of customer (VOC) [46, 47], usability testing (thinking aloud protocol) [48, 49], Kansei engineering [50, 51], or quality function deployment (QFD) [52–54] are systematically utilized. Innovation science using function-object analysis [55, 56] or main parameter value [57] is also important to mention. For the conceptual design phase of innovation process is suitable to use modern creative techniques supporting idea generation and overcoming technical and physical contradictions based on TRIZ [58–61]. The process of concept generation is finished by choosing between concept alternatives by simple evaluation charts [28] or advanced techniques or analytic hierarchy process [4]. Since the above methods are becoming standards when upgrading technical products in 21st century it is clear that further development and innovation of machine for mechanical extraction of oil from* Jatropha curcas *L. require similar advanced techniques and methods.

## 2. Complex Approach to Design Specification and Concept Generation

Generation of the new technical product concept is in general carried out in the following two steps:

 1st step: target design specification determining
 2nd step: system or components concept(s) generation

**Figure 1:**
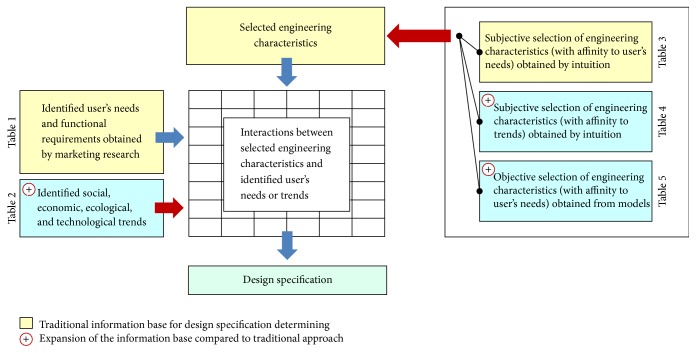
Complex approach to design specification determining with expanded information base.

In the first step the information transformations described in the Introduction take place. Abovementioned approaches [42, 44–54] to these transformations of needs to design specification are often focused only on the transformation of the primary needs formulated intuitively and subjectively by users during marketing research or on the evaluation of attributes of competing products (Figure 1).

In today's world of technology, which leads to accelerated development, for example, in technologies or material science, to include to the mentioned transformations only information from users (farmers) or information about other similar products is insufficient. That is because users do not have and cannot have sufficient knowledge of the possibilities of current technologies or have not access to information about trends in the relevant fields of technology. Users (farmers) can only guess at what is possible in present and near future design. For that reason it is necessary to enrich traditional approach to determination of the design specification. First technological, ecological, economic, and social trends should be included in a set of functional requirements (needs)—Figure 1. Second relevant engineering characteristics with affinity to technological, social, or economic trends should be involved into process of design specification determining as well (Figure 1). Third designers should additionally include information obtained by modelling that can objectively on the basis of physical and chemical laws extend set of suitable engineering characteristics describing future machine (Figure 1).

After design specification determining logical process of problem solving or progressive techniques supporting designer's creativity should be used to obtain a concept of innovative solution. Among these techniques we can include

- function oriented search [62] or function-behaviour oriented search [63],
- 40 inventive principles and heuristics [64, 65],
- laws or trends of evolution of technical systems [66, 67],
- multilevel system thinking [68, 69],
- morphological matrix [70] or solution catalogue [71].

Utilization of the described complex approach to design specification and concept generation will be partially demonstrated on machine extracting oil from* Jatropha curcas *L. seeds for small-scale producers or farmers.

## 3. Case Study: Conceptual Design of Machine Mechanically Extracting Oil from* Jatropha curcas *L. Seeds in Small-Scale

**Figure 2:**
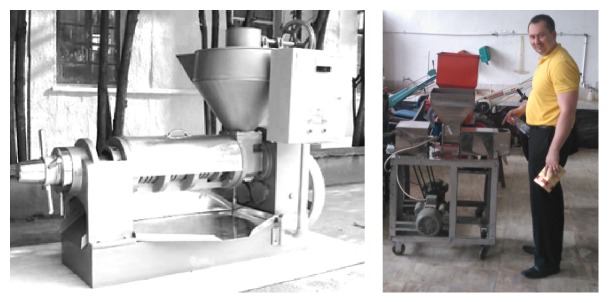
Typical machines mechanically extracting oil from* Jatropha curcas *L. seeds.

Basic functions of the mechanical extraction machine consist in separating the solid component (structures) and liquid component (oil). Linear or nonlinear pressing (vertical, horizontal, or angled) by a sliding piston or rotary screw is frequently used for small-scale production. As the technological set-up for* Jatropha* processing is not yet fully developed and progress may be made in terms of mechanisation [15] we present mentioned complex approach in the following case study focused on conceptual design of screw extractor press extracting biooil from* Jatropha curcas *L. seeds for small-scale production. First, the research team analysed sources [7–9, 13–17, 19] and information obtained during interview realized in Sumatra and Java (Indonesia)—Figure 2. Low production cost [14], high productivity [16], and higher oil yield [19] were considered as essential extracting machines user's needs (Table 1).

In accordance with the principles described in Section 2 (Figure 1) the set of primary user's needs was subsequently extended by trends in the production of oil and biofuel from* Jatropha curcas *L. At extraction machine attributes determining process not only the primary user's needs, but also “unexpected” needs based on technological, economic, social, or ecological trends were taken into account. By the research of [7, 16, 18, 19] and by interviews made in Indonesia we identified the following trends:

- Trend to higher degree of cleaning of crude oil from solid particles (husks) [7, 19]
- Trend to lower oil content in the seed cake [8]
- Trend to necessary competition with big corporations [16]
- Trend to commercial use of the seed cake after oil extraction [18]
- Trend to mechanical separating kernels and husks [19]
- Trend to more profitable technology [19]

Interpretation of these trends upon their integration is shown in Table 2.

The next step in the process of design specification determination was to select appropriate engineering characteristics with affinity to identified primary user's needs and trends. First measurable engineering characteristics of the innovated extraction machine with affinity to interpreted user's needs were subjectively and intuitively selected by traditional approach (Table 3).

Consequently measurable engineering characteristics of the innovated machine for oil extraction from* Jatropha curcas *L. with affinity to interpreted trends were subjectively and intuitively selected (Table 4).

**Figure 3:**
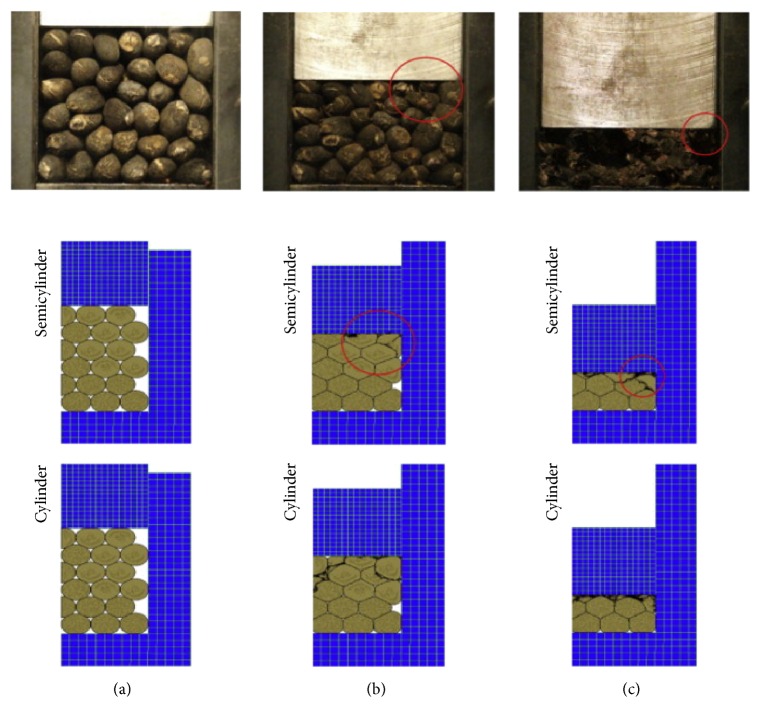
FEM model for pressing the seeds in a container [24].

**Figure 4:**
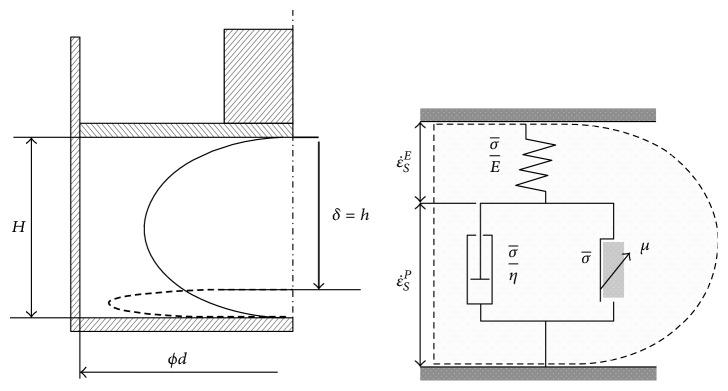
Perzyna model for the study of the deformation and compression of seed [23].

**Figure 5:**
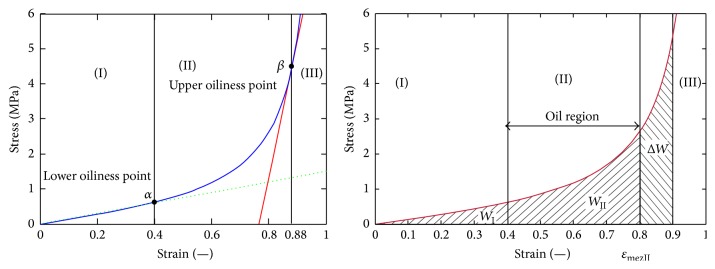
Oil region for maximum oil yield [72].

**Figure 6:**
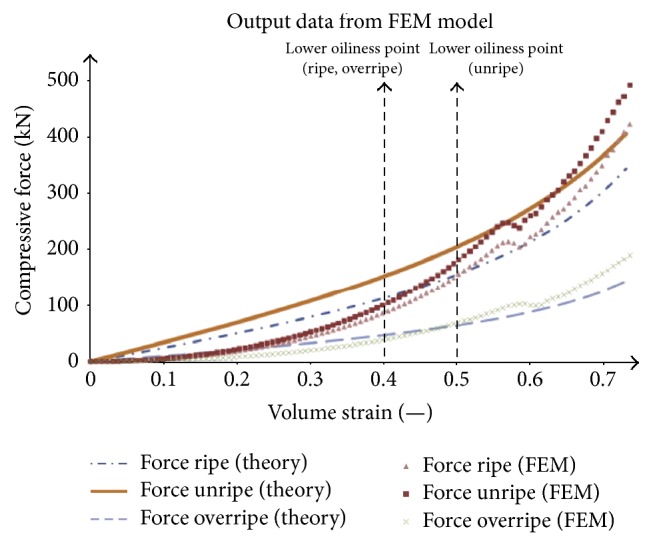
Determination of compressive force to press the seed (ripe, unripe, and overripe) [23].

**Figure 7:**
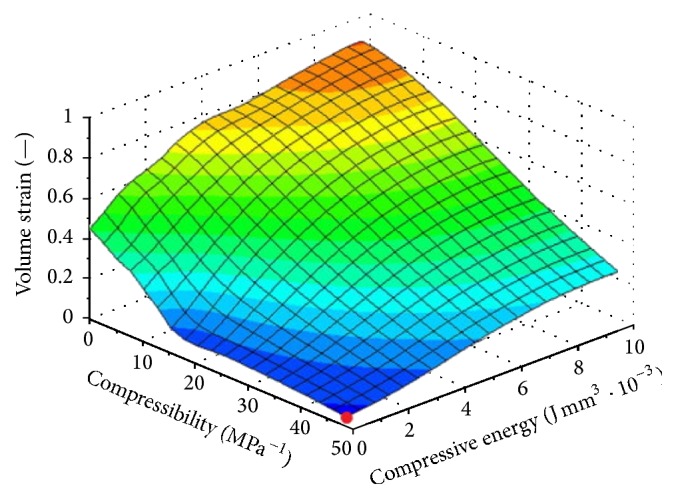
Parametric representation of the volume strain, the compressive energy, and volume compressibility during pressing seeds [24].

As mentioned in Section 2, it is important to include phenomena occurring during oil extraction into process of design specification setting (Figure 1). Intuitively and subjectively selected engineering characteristics listed in Tables 3 and 4 were subsequently extended by set of characteristics obtained during an objective study of appropriate physical and mathematical models describing relevant phenomena and processes. In that case we use the following models:

 Model Nr. 1: model for pressing the seeds in a container (Figure 3)
 Model Nr. 2: Perzyna model for the study of the deformation and compression of seed (Figure 4)
 Model Nr. 3: determination of oil region for maximum oil yield (Figure 5)
 Model Nr. 4: determination of compressive force to press different state seeds (Figure 6)
 Model Nr. 5: representation of the volume strain, the compressive energy, and volume compressibility during seeds pressing (Figure 7)

It is known that the volumetric strain and deformation of seeds increase the stress and energy intensity. Petrů et al. and Herak et al. [24, 72] set bound of oil region between lower oiliness point (LOP = 40%) and upper oiliness point (UOP = 80%)—Figure 5. From Figures 5 and 6 it is apparent that the increase in oil yield is possible by increasing the compressive force.

Based on the analysis of enumerated physical and mathematical models other important engineering characteristics were identified. These characteristics used for the complex approach to design specification determination are summarized in Table 5.

**Figure 8:**
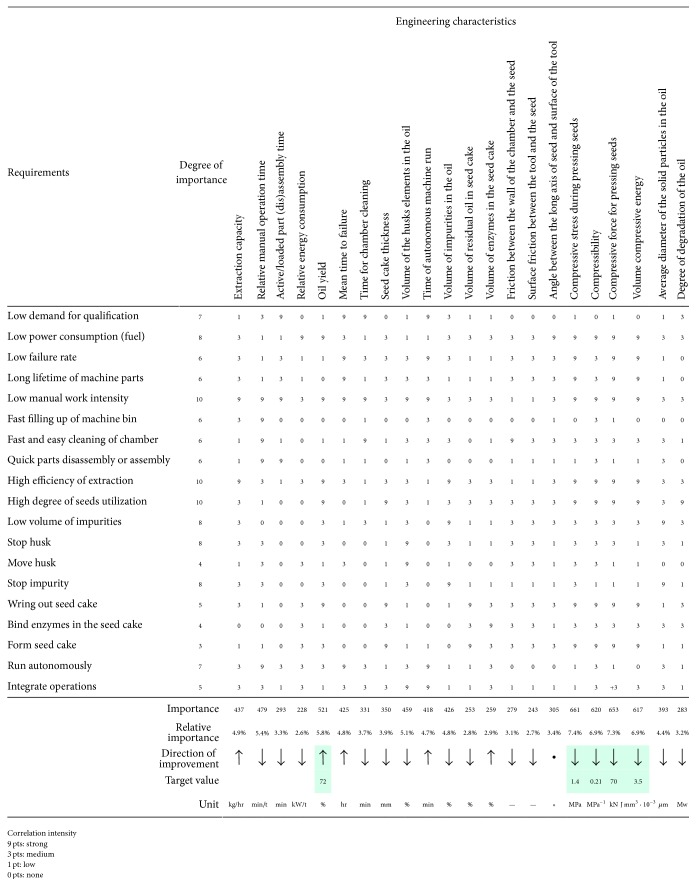
Correlation matrix used for design specification of machine mechanically extracting oil from* Jatropha curcas *L. seeds.

The number of identified engineering characteristics (Tables 3, 4, and 5) was then reduced by characteristics of integration (e.g., silo and tank volume and autonomous machine run) or by review of their affinity to identified requirements. Selected characteristics should have to ensure that the new innovated machine will better satisfy small-scale producers or farmers. In accordance with the complex approach described in Section 2 correlation matrix for detection and evaluation of interactions between engineering characteristics and requirements (interpreted needs or trends) was prepared (Figure 8). This matrix indicates how engineering characteristics affect the satisfaction of each requirement. Compared to the standardized QFD method [52–54] a comparison with competing products both in terms of information from the market and in the context of comparing engineering characteristics has not been conducted. The degree of importance (1 = min, 10 = max) of small-scale farmers individual requirement was based on expert subjective opinion of research team members which is the usual procedure.

The significance of individual engineering characteristics was calculated by standard manner as the sum of the products of the degree of importance of requirement and correlation intensity (e.g., strong, medium, weak, or nonexistent). The resulting correlation matrix (Figure 8) showed that in terms of the functional requirements imposed on the machine mechanically extracting oil from seeds of* Jatropha curcas *L. the most important engineering characteristics are

- compressive stress during pressing seeds (absolute importance 661),
- compressive force for pressing seeds (absolute importance 653),
- compressibility (absolute importance 620),
- volume compressive energy (absolute importance 617),
- oil yield (absolute importance 551).

These important engineering characteristics were considered by the research team as the essential and relevant target numeric values were set as design specification for upgrading the machine mechanically extracting oil from* Jatropha curcas *L. seeds. As the design of the proper screw (chamber) plays the most important role in the oil extraction efficiency we will pay in next part of the paper attention to the new concept of screw design.

**Figure 9:**
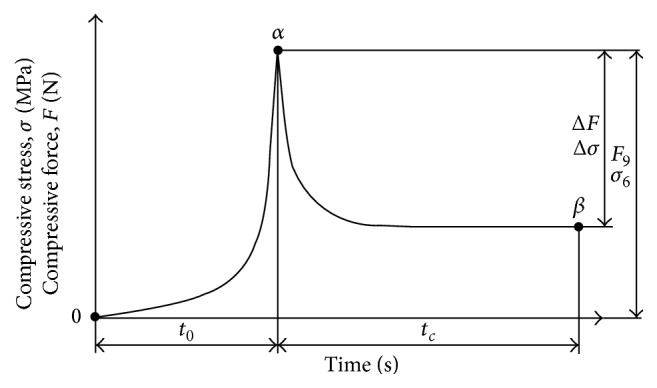
Compression and relaxation during oil seeds pressing [72].

**Figure 10:**
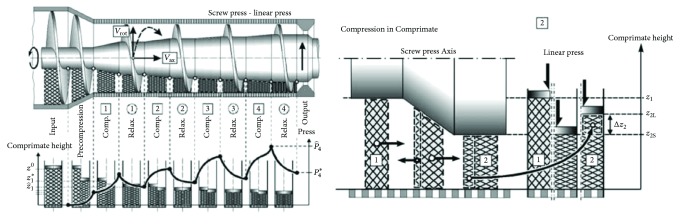
Variable geometry of compression chamber geometry and screw [73].

It is known [72] that the process of oil extraction from* Jatropha curcas *L. seeds can be streamlined by using changes of pressing force at a time, that is, by compression and subsequent relaxation (Figure 9). This can be technically ensured by designing the various pressing chambers known [73] and by changing the geometry of the screw as shown in Figure 10. The distribution of oil yield in individual chambers of the press machine is also illustrated in Figure 10.

**Figure 11:**
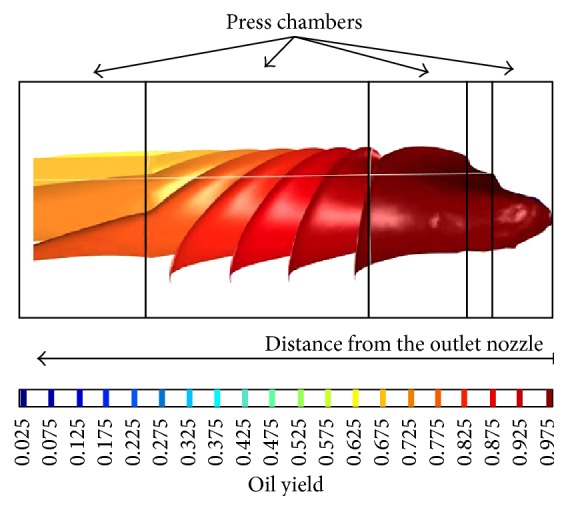
Visualization of the oil yield depending on the compression volume of chambers.

**Figure 12:**
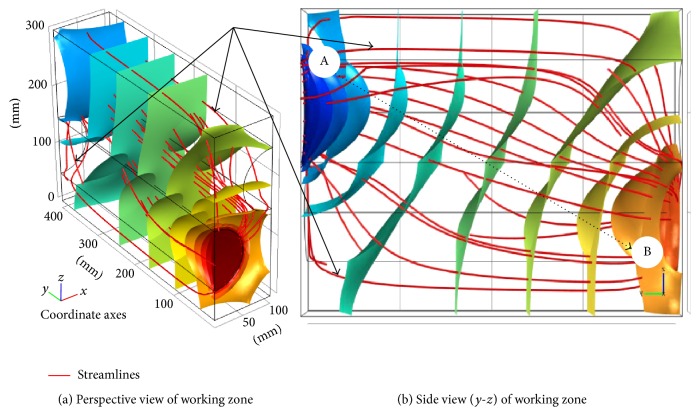
Numerical model—visualization of the maximum compressive energy required in the oil extracting machine depending on the compression volume of chamber (A—area with the lowest compressive energy and stress distribution in the working zone of oil extracting machine; B—area with the highest compressive energy and stress distribution in the working zone of oil extracting machine).

However, the problem arises due to enormous rise of temperature caused by the friction among seeds, chamber walls, and tool and especially due to increase of the compression energy required for the extraction of oil regarding the maximum compressive space in maximum recovery of oil (Figures 11 and 12).

For assessment and visualization of maximum theoretical volumetric oil yield the numerical model was created (Figure 11). The model shows the unit distribution of oil yield (max = 1, min = 0) in the individual chambers of the press, whose design is similar to press in Figure 10. The model illustrates one of the principal problems of mechanical oil pressing that occurs when the compression energy necessary for extruding the oil is increasing beyond a certain limit. According to the presented numerical model (Figure 11) it is ideally apparent that minimum oil yield can be achieved in the chambers with a lower filling and vice versa maximum oil yield (97.5%) can be achieved in the chambers with a higher filling. In real conditions of oil extraction this maximal oil yield is not achieved due to the fact that standard extraction presses are not able to provide the required compression energy. The reason for this oil yield limitation is that forces and stress exponentially increase in relation to the compression (Figures 5 and 6). From the point of view of oil extraction efficiency a new concept of machine mechanically extracting oil from* Jatropha curcas *L. seeds should therefore solve technical problems rising in the chamber with the largest filling where compressive stress and compressive energy are the highest. Unequal loading of the individual chambers of the oil extracting machine is also seen from streamlines courses in Figure 12. Low compression energy will be generated on the left side of the working zone (area A in Figure 12), while the highest compression energy will be generated on the right side of the working zone close to the outlet nozzle of oil extracting machine (area B in Figure 12).

Not only do the above phenomena cause technical problems (maximum force acting on parts of the machine), but increased temperature also causes oil degradation, change in its chemical composition, and the subsequent quality deterioration of the oil for the production of biofuel. Due to these phenomena design of the extraction machine screw and chamber shown in Figure 10 cannot be fully used and therefore maximum oil recovery cannot be achieved by this solution (we gain yield around 40–65%).

To solve this technical problem we used the following two techniques of innovation science:

- Trends of evolution of technical systems—statistically authentic evolution lines describing the regular evolutionary transition of systems from one specified state to another, being true for all technical (engineering) systems [58].
- Inventive principles—each inventive principle represents concept or idea that may be applied to solve the problem situation; it means to remove technical contradiction [64, 68]. Technical contradiction is formed when there is an obstacle to making an improvement.

Using these techniques following directions of solution have been identified:

- Asymmetry (symmetry change): change the shape or properties of an object from symmetrical to asymmetrical or change the shape of an object to suit external asymmetries.
- Dynamics: change the object (or outside environment) for optimal performance at every stage of operation, divide an object into parts capable of movement relative to each other, or increase the degree of free motion.
- Another dimension: move into an additional dimension—from one to two—from two to three or use the other side.
- Periodic action: instead of continuous action, use periodic or pulsating action.
- Porosity: make an object porous or add porous elements.

**Figure 13:**
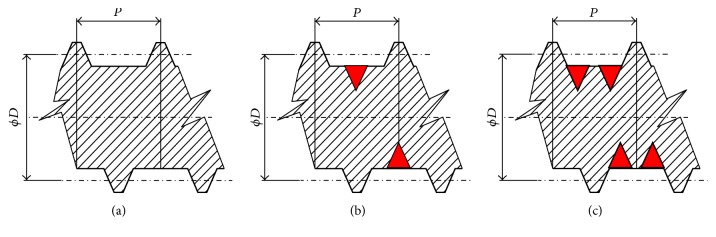
Traditional solution of screw and new concept of screw for machine mechanically extracting oil from* Jatropha curcas *L. seeds: (a) standard screw design, (b) screw design with one groove for decrease of compressive force and expended energy reduction, and (c) screw design with two grooves for decrease of compressive force and expended energy reduction.

**Figure 14:**
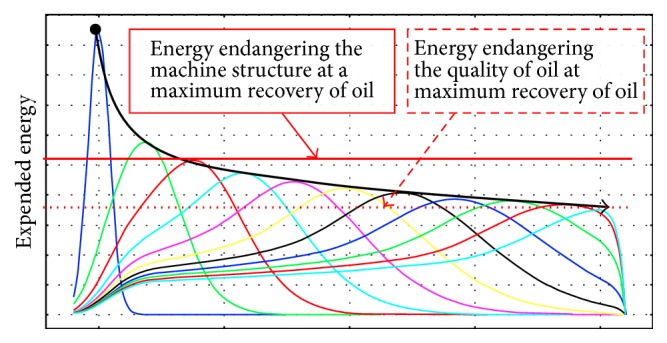
Courses of expended energy using the original screw geometry (dark blue) and using the new design concept with modified geometry of the screw with one groove in multiple variations of groove shape (other colours).

**Figure 15:**
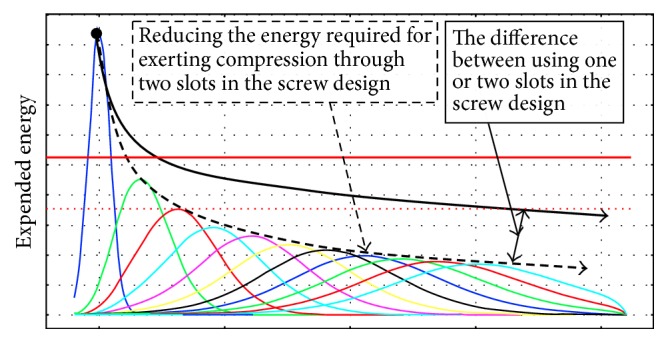
Courses of expended energy using the original screw geometry (dark blue) and using the new design concept with modified geometry of the screw with two grooves in multiple variations of groove shape (other colours).

With creative support of these heuristics a concept of solution of mentioned problem was prepared. Problem and cause of lower oil yield could be resolved by automatically opening one or more grooves for discharging solid particles (hulks, impurities) in the body of the screw (Figure 13). The grooves would be opened when compressive energy is high and the grooves would be closed automatically when the compressive power is under certain level. A conceptual comparison of traditional screw design and two variants of innovated screw is illustrated in Figure 13. Effects of presented new variants of screw geometry and the number of grooves are shown in Figures 14 and 15.

## 4. Conclusions

Big attention should be paid to the systematic design of machines used in the process-chain for biomass fuel production. Without a more complex approach to design specification and concept generation machines for mechanical extracting oil from seeds (biospecies) cannot accomplish the necessary efficiency or productivity and in particular small-scale producers cannot achieve desired profitability. It is useful to include extended information base into determination of design specification. Standard information base should be enriched by technological, ecological, economic, and social trends and by information obtained by modelling that can objectively on the basis of physical and chemical laws extend set of suitable engineering characteristics describing parameters of future machines. In the case of machine mechanically extracting oil from* Jatropha curcas *L. seeds concept generation known and entirely new models were employed. These models refined original theoretical considerations and were consequently used to illustrate the relationship between compression force, compression energy, and new shapes of extraction machine screw. Using the model shows new possibilities for improving the pressing process towards a maximum yield of oil for biomass-based fuel production. It was also confirmed that it is appropriate and necessary to use advanced tools of innovation science as inventive principles or trends of evolution of technical systems. By the complex approach to design specification and concept generation the new concept of screw shape was proposed. Since presented case study shows the big potential in terms of utilization modelling and innovation science techniques it is necessary to continue with focused research to improve machinery performance and thus support small-scale farmers or biomass-based fuel produces.

## Figures and Tables

**Table 1:** Decomposition and interpretation of primary user's needs (machines mechanically extracting oil).

Primary user's need	Need decomposition	Interpretation of need (requirement)
Low operational cost	Operated by unskilled worker	Low demand for qualification
	Low energy consumption	Low power (fuel) consumption
	High availability	Low failure rate
	Long lifetime	Long lifetime of machine parts

High productivity	High labour productivity	Low manual work intensity
	Fast filling up of machine	Fast filling up of machine bin
	Fast and easy cleaning	Fast and easy cleaning of chamber
	Easy spare parts replacement	Quick parts disassembly or assembly

High yield of quality oil	High oil yield	High efficiency of extraction
	Low waste	High degree of seeds utilization
	Clean oil	Low volume of impurities

**Table 2:** Decomposition and interpretation of identified trends.

Identified trend	Trend decomposition	Interpretation of trend (requirement)
Husks removal	Husks separation	Stop husk
	Husks removing	Move husk
	Impurities separation	Stop impurity

Better utilization of seed cake	Obtain oil from seed cake	Wring out seed cake
	Control of the seed cake composition	Bind enzymes in the seed cake
	Seed cake processing	Form seed cake

More profitable oil extraction	Reduce operational costs	Run autonomously
	Integrate activities	Integrate operations

**Table 3:** Selected engineering characteristics with affinity to interpreted user's needs.

Engineering characteristic	Unit
Extraction capacity	kg/hr
Relative manual operation time	min/t
Seeds silo volume	m^3^
Oil tank volume	m^3^
Active/loaded part (dis)assembly time	min
Relative energy consumption	kW/t
Oil yield	%
Mean time to failure	hr
Time for chamber cleaning	min

**Table 4:** Selected engineering characteristics with affinity to interpreted trends.

Engineering characteristic	Unit
Seed cake thickness	mm
Volume of the husks elements in the oil	%
Time of autonomous machine run	min
Volume of impurities in the oil	%
Volume of residual oil in seed cake	%
Volume of enzymes in the seed cake	%

**Table 5:** Engineering characteristics determined by analysis of physical and mathematical models.

Engineering characteristic	Unit
Friction between the wall of the chamber and the seed	—
Surface friction between the tool and the seed	—
Angle between the longitudinal axis of the seed and the surface of the tool	°
Compressive stress during pressing ripe seeds	MPa
Compressibility	MPa^−1^
Compressive force for pressing seeds	kN
Volume compressive energy	*J* mm^3^ · 10^−3^
Average diameter of the solid particles in the oil	*μ*m
Degree of degradation of the oil	Mw
